# Advances and Prospects in Using Induced Pluripotent Stem Cells for 3D Bioprinting in Cardiac Tissue Engineering

**DOI:** 10.31083/RCM26697

**Published:** 2025-03-19

**Authors:** Baoluo Du, Ziqiang Dai, Huan Wang, Zhipeng Ren, Dianyuan Li

**Affiliations:** ^1^Department of Cardiovascular Surgery, The Affiliated Suzhou Hospital of Nanjing Medical University, Suzhou Municipal Hospital, Gusu School, Nanjing Medical University, 215008 Suzhou, Jiangsu, China

**Keywords:** cardiac tissue engineering, bio-3D printing, stem cells, tissue engineering scaffold, induced pluripotent stem cells, iPSCs

## Abstract

**Background:**

Cardiovascular diseases remain one of the leading causes of death worldwide. Given the limited self-repair capacity of cardiac tissue, cardiac tissue engineering (CTE) aims to develop strategies and materials for repairing or replacing damaged cardiac tissue by combining biology, medicine, and engineering. Indeed, CTE has made significant strides since the discovery of induced pluripotent stem cells (iPSCs) in 2006, including creating cardiac patches, organoids, and chip models derived from iPSCs, thus offering new strategies for treating cardiac diseases.

**Methods:**

A systematic search for relevant literature published between 2003 and 2024 was conducted in the PubMed and Web of Science databases using “Cardiac Tissue Engineering”, “3D Bioprinting”, “Scaffold in Tissue Engineering”, “Induced Pluripotent Stem Cells”, and “iPSCs” as keywords.

**Results:**

This systematic search using the abovementioned keywords identified relevant articles for inclusion in this review. The resulting literature indicated that CTE can offer innovative solutions for treating cardiac diseases when integrated with three-dimensional (3D) bioprinting and iPSC technology.

**Conclusions:**

Despite notable advances in the field of CTE, multiple challenges remain relating to 3D-bioprinted cardiac tissues. These include maintaining long-term cell viability, achieving precise cell distribution, tissue vascularization, material selection, and cost-effectiveness. Therefore, further research is needed to optimize printing techniques, develop more advanced bio-inks, explore larger-scale tissue constructs, and ensure the biosafety and functional fidelity of engineered cardiac tissues. Subsequently, future research efforts should focus on these areas to facilitate the clinical translation of CTE. Moreover, additional long-term animal models and preclinical studies should be conducted to ensure the biosafety and functionality of engineered cardiac tissues, thereby creating novel possibilities for treating patients with heart diseases.

## 1. Introduction

Cardiac tissue engineering (CTE) combines knowledge and technologies from biology, medicine, and engineering to develop and implement solutions for repairing and regenerating damaged cardiac tissue. Given the increasingly heavy burden of cardiovascular diseases worldwide, CTE research has become particularly urgent [1]. Cardiovascular diseases remain one of the leading causes of death globally, according to data from the World Health Organization, causing approximately 17.9 million deaths annually, with 85% of these deaths from heart attacks and strokes. In Asia, cardiovascular diseases account for 30% of total deaths, while in Europe, this proportion is even higher, reaching 45%. The annual incidence of myocardial infarction (MI) is estimated to be 790,000 cases, with coronary artery disease responsible for 1 in 7 deaths [2, 3]. These statistics highlight the importance of researching methods to prevent and treat cardiovascular diseases; developing novel CTE strategies holds significant promise for improving patient outcomes. The dedicated focus of CTE is to create and implement strategies for repairing and regenerating damaged cardiac tissue. Indeed, CTE research has progressed significantly since the landmark breakthrough in developing induced pluripotent stem cell (iPSC) technology in 2006, which has successfully created complex cardiac tissue structures based on iPSCs, such as cardiac patches, organoids, and chip models. These advancements can provide new insights into the pathogenesis of heart disease and further create newer therapeutic strategies [2, 4].

Research in CTE has attracted significant attention in recent years in its attempt to address challenges in treating cardiovascular diseases. For example, the heart loses approximately one billion cardiomyocytes (CMs) during acute myocardial infarction. Given the extremely limited regenerative capacity of CMs, the injection of cells at five to six sites within and around the infarcted area has been used in an attempt to restore cardiac function and compensate for lost myocardial tissue [5]. However, multiple injections of large volumes of cells into the infarcted region can lead to uneven cell distribution and an increased risk of ventricular arrhythmias [6]. Therefore, researchers have turned to CTE to develop cardiac patches that deliver many cells uniformly over the area of myocardial damage, thereby achieving better therapeutic outcomes [7]. However, existing CTE technologies have certain limitations affecting treatment efficacy, such as the risk of immune rejection when engineered tissue is implanted in the human body [8]. Therefore, thick, multi-layer muscular tissue is required to achieve clinical benefit, yet the maximum distance for nutrient/oxygen diffusion to occur successfully without vascularization is approximately 100–200 µm [9]; numerous studies have confirmed these limitations.

While current medical materials have achieved remarkable results in clinical applications, they still suffer from limitations such as thrombogenic reactions, immune rejection, and durability issues [10, 11]. As a result, material innovation has become a major area of study in current research, with the ultimate goal of developing fully functional medical materials for repairing or replacing damaged tissues [12]. This necessitates comprehensive investigation, from optimizing cell sources and culture environments designing biomaterial scaffolds, applying bioprinting technologies, constructing tissues, and modulating biological signals. Notably, combining three-dimensional (3D) bioprinting technology with the directed differentiation of stem cells has enabled the construction of functional cardiac tissues, thus showcasing the significant potential for promoting tissue regeneration and enhancing cardiac function [13]. However, traditional repair techniques, such as conventional stem cell injection, offer less precise control over cell distribution than 3D bioprinting technology. Further, traditional stem cell injection often leads to uneven cell distribution and difficulty controlling long-term cell survival in the damaged area [6]. In contrast, 3D bioprinting technology, through precise control of the three-dimensional distribution of cells, can better mimic the structure of natural cardiac tissue, thus improving cell survival rates and tissue function.

Hence, to overcome these limitations in the current materials, researchers are exploring strategies such as using materials with improved immune compatibility, developing more refined cell delivery systems, and optimizing cellular microenvironments [14]. These efforts promise to improve the future clinical outcomes of CTE applications, yet despite remarkable achievements, CTE still faces a series of technical challenges. These include maintaining the long-term viability of printed cells, achieving precise cell positioning, efficient vascularization of tissues, and optimizing material selection and cost-effectiveness. Thus, researchers are currently concentrating their efforts on four main areas to enhance the clinical application of CTE: refining 3D printing processes, developing more advanced bio-inks, exploring tissue construction protocols, and conducting long-term safety and functionality validation [15].

This review systematically evaluates existing CTE technologies and compares the advantages and limitations of different techniques. Furthermore, it covers the progress of 3D bioprinting in CTE and discusses its future development, thus providing a theoretical basis and practical guidance for the clinical application of CTE.

**Fig. 1.**
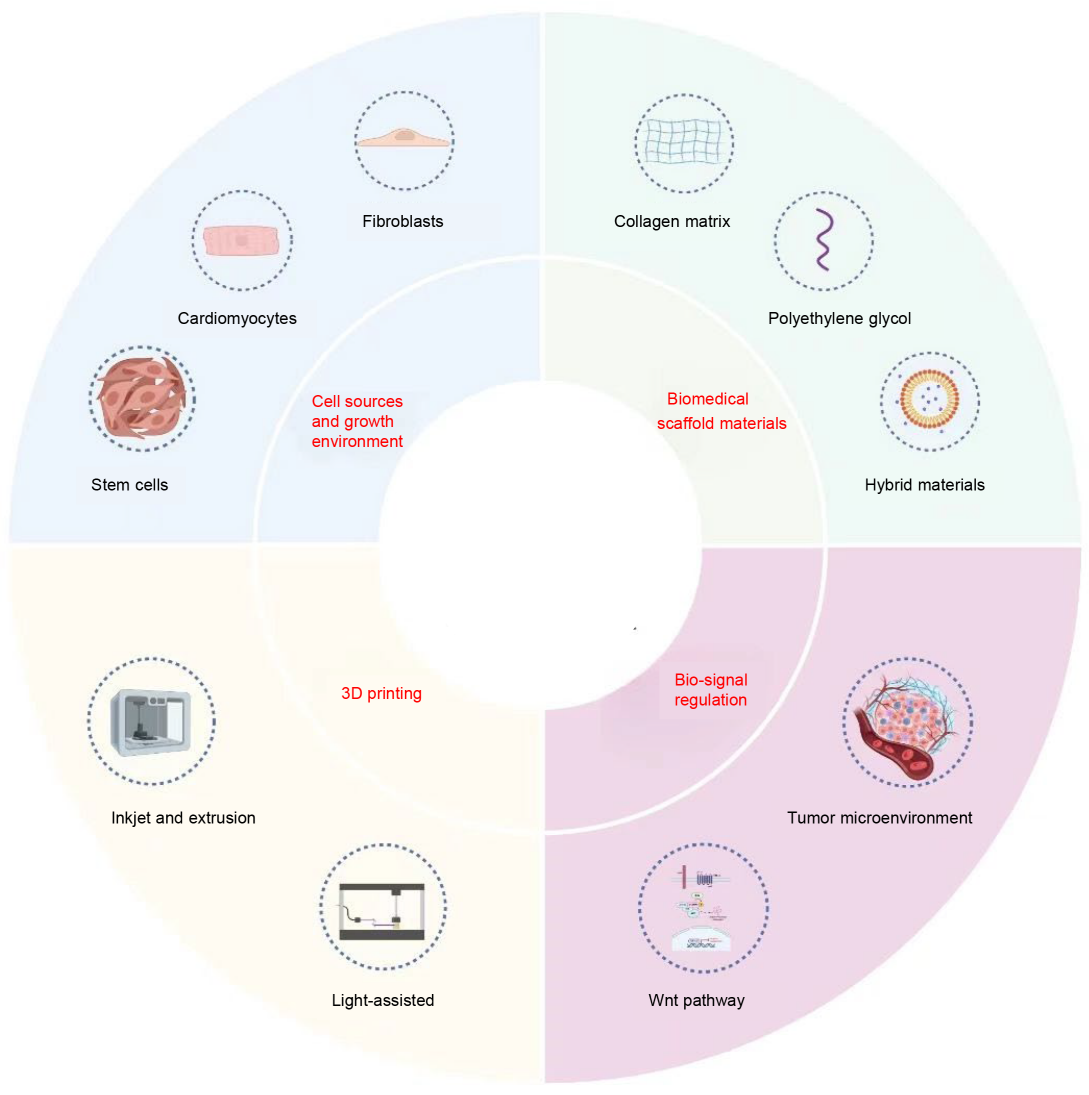
**Materials and methods used in cardiac tissue engineering**. 3D, three-dimensional. The Figure is created by BioRender.

Fig. 1 shows the interrelation and application of the four major areas (in red font) involved in CTE.

## 2. Materials and Methods

This literature search was conducted according to the methodological framework proposed by the PRISMA (Preferred Reporting Items for Systematic Reviews and Meta-Analyses) guidelines. The search was performed using the PubMed and Web of Science databases in April 2024 with the terms “Cardiac Tissue Engineering”, “3D Bioprinting”, “Scaffold in Tissue Engineering”, “Induced Pluripotent Stem Cells”, and “iPSCs” in both subject headings and as keywords. The retrieval time range was set from 2003 to 2024. Additionally, the reference lists in the retrieved articles were manually searched to supplement the sources. Following the literature search, two independent evaluators reviewed and screened all identified records according to the predefined inclusion and exclusion criteria. When discordances arose between the evaluators, these were resolved through discussion or consultation with a third evaluator. Inclusion criteria encompassed studies involving research and applications in CTE, while exclusion criteria included conference abstracts, editorials, reviews, and commentary articles. The collected data included the first author’s name, year of publication, study objectives, 3D printing methods, animal experiments, bio-inks, cell viability, and printed models. To ensure transparency and completeness of the systematic review throughout the process, the requirements of the PRISMA checklist were strictly adhered to.

### Quality Appraisal

The Checklist of Review Criteria was used in the final review library as specified by the Task Force of Academic Medicine and GEA-RIME (the Group on Educational Affairs-Research in Medical Education) committee to assess the quality and relevance of the papers instead of evaluating the title and abstract alone [16]. Using this framework, each section of the identified publications can be assessed for scientific merit and features that should remain consistent throughout the paper, such as well-identified research problems, robust experimental design, and critical data analysis. The categories within the Checklist of Review Criteria are as follows, with the corresponding numbers matching those in Table 1 (Ref. [17, 18, 19, 20, 21, 22, 23, 24, 25, 26, 27]):

1. Problem statement, conceptual framework, and research question

2. Reference to the literature and documentation

3. Relevance

4. Research design

5. Instrumentation, data collection, and quality control

6. Population and sample

7. Data analysis and statistics

8. Reporting of statistical analyses

9. Presentation of results

10. Discussion and conclusion: interpretation

11. Title, authors, and abstract

12. Presentation and documentation

13. Scientific conduct

**Table 1.** **Quality appraisal of the 11 publications retrieved after database inception and search attrition**.

Checklist of Review Criteria Categories														
Publication	1	2	3	4	5	6	7	8	9	10	11	12	13	Total criteria met
Arai *et al*. [17]	√	√	√	√	√	√	√	√	√	√	√	√	√	13
Maiullari *et al*. [18]	√	√	√	√	√	√	√	√	√	√	√	√	√	13
Anil Kumar *et al*. [19]	√	√	√	√	√	√	√	√	√	√	√	√	√	13
Yu *et al*. [20]	√	√	√	√	√	√	√	√	√	√	√	√	√	13
Yeung *et al*. [21]	√	√	√	√	√	√	√	√	√	√	√	√	√	13
Lou *et al*. [22]	√	√	√	√	√	√	√	√	√	√	√	√	√	13
Noor *et al*. [23]	√	√	√	√	√	√	√	√	√	√	√	√	√	13
Pretorius *et al*. [24]	√	√	√	√	√	√	√	√	√	√	√	√	√	13
Tsukamoto *et al*. [25]	√	√	√	√	√	√	√	√	√	√	√	√	√	13
Miller *et al*. [26]	√	√	√	√	√	√	√	√	√	√	√	√	√	13
Sridharan *et al*. [27]	√	√	√	√	√	√	√	√	√	√	√	√	√	13
Total papers														11

(1) Problem statement, conceptual framework, and research question; (2) reference to literature and documentation; (3) relevance; (4) research design; (5) instrumentation, data collection, and quality control; (6) population and sample; (7) data analysis and statistics; (8) reporting of statistical analyses; (9) presentation of results; (10) discussion and conclusion: interpretation; (11) title, authors, and abstract; (12) presentation and documentation; (13) scientific conduct.

## 3. Results

**Fig. 2.**
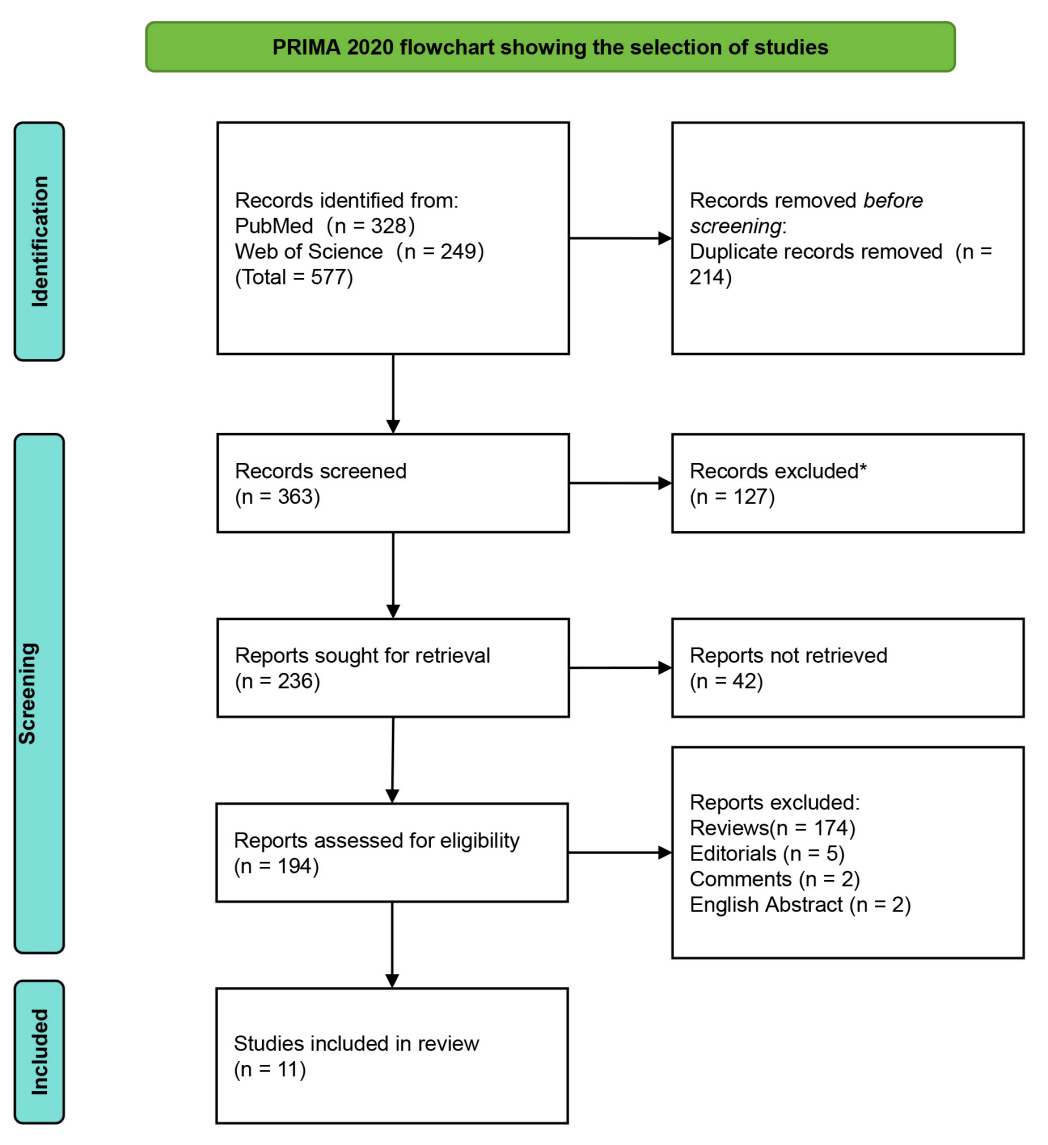
**PRISMA flowchart**. *Reasons for exclusion included:publications must be; primary research, in English and open access. PRISMA, Preferred Reporting Items for Systematic Reviews and Meta-Analyses.

The PubMed and Web of Science database searches identified 328 and 249 studies, respectively. According to the inclusion criteria, articles had to be published after 2018, have high relevance, and use iPSC-derived cells; meanwhile, the exclusion criteria removed articles that were not original research, in English, or open access; this process ultimately identified 11 highly relevant articles for analysis, which were evaluated using 13 criteria. Papers not meeting at least 12 criteria were excluded from further consideration (see Table 1). All publications passed the quality assessment process and were deemed suitable for inclusion in this systematic review. Fig. 2 illustrates the literature search results conducted according to the PRISMA guidelines.

### 3.1 Cell Sources and Growth Environment

One of the core challenges in CTE is obtaining sufficient and functional cell sources for constructing or repairing cardiac tissue. Currently, the most often used cells in research include stem cells such as iPSCs, CMs, and fibroblasts, essential for recreating cardiac tissue structure and function [28, 29, 30]. By simulating the cardiac microenvironment* in vitro*, the directed differentiation and proliferation of cells can be effectively promoted, leading to the formation of complex multi-cellular structures [31, 32].

The discovery and application of iPSCs have significantly advanced the field of CTE [33]. Since the initial success of the Yamanaka laboratory in 2006 in reprogramming adult cells into iPSCs [29], this technology has navigated the ethical controversies associated with using embryonic stem cells (ESCs), creating many possibilities for personalized medicine and drug screening [34]. Table 2 (Ref. [6, 8, 35, 36, 37, 38, 39, 40]) summarizes the selection criteria for the different cell types used in CTE and their respective advantages and disadvantages. Initially, the efficiency of generating iPSCs was low. However, the reprogramming efficiency has been significantly improved through continuous research and innovation, especially by utilizing specific compounds (such as valproic acid, sodium butyrate, and histone deacetylase inhibitors) and by optimizing the culture conditions (such as hypoxic environments and appropriate medium) [41, 42, 43, 44]. Moreover, to overcome the issue of genomic integration, which is common in traditional iPSC culture methods, researchers have developed various non-integrating approaches, including adenoviruses, plasmid vectors, and Sendai viruses. Additionally, using combinations of small molecules has sometimes enabled mouse embryonic fibroblasts to be reprogrammed into iPSCs without requiring genetic manipulation [45].

**Table 2.** **Cell sources used in cardiac tissue engineering: advantages and disadvantages**.

Classification	Source	Advantages	Disadvantages
Cardiac stem cells (CSCs) [35]	Heart	Since they are derived from cardiac tissue, CSCs have the potential to differentiate into specialized cardiac cells, such as CMs and endothelial cells	Heart muscle tissue acquisition; low induction differentiation rate; difficulty of expanding *in vitro*
Mesenchymal stem cells (MSCs) [36]	Bone marrow, adipose tissue	Readily available source; easy to isolate, cultivate, and expand; has immune regulatory capability; has high transplantation safety	Weak ability for direct differentiation into CMs; limited differentiation potential that decreases with the number of passages; the third generation shows better differentiation efficiency
Embryonic stem cells (ESCs) [6, 8, 37]	Inner cell mass of blastocyst-stage embryos	High potential for differentiation; able to differentiate into all cell types in the heart; relatively abundant source	Procurement involves ethical issues; risks of teratoma formation and arrhythmias; risk of immune rejection upon transplantation
Induced pluripotent stem cells (iPSCs) [38, 39, 40]	Reprogrammed mature somatic cells	Have the advantages of ESCs; autologous procurement avoids ethical controversies; easy to obtain; suitable for autologous transplantation; reduces the risk of immune rejection	iPSCs-derived CMs have low maturity; risks of teratoma formation and arrhythmias

CMs, cardiomyocytes.

**Fig. 3.**
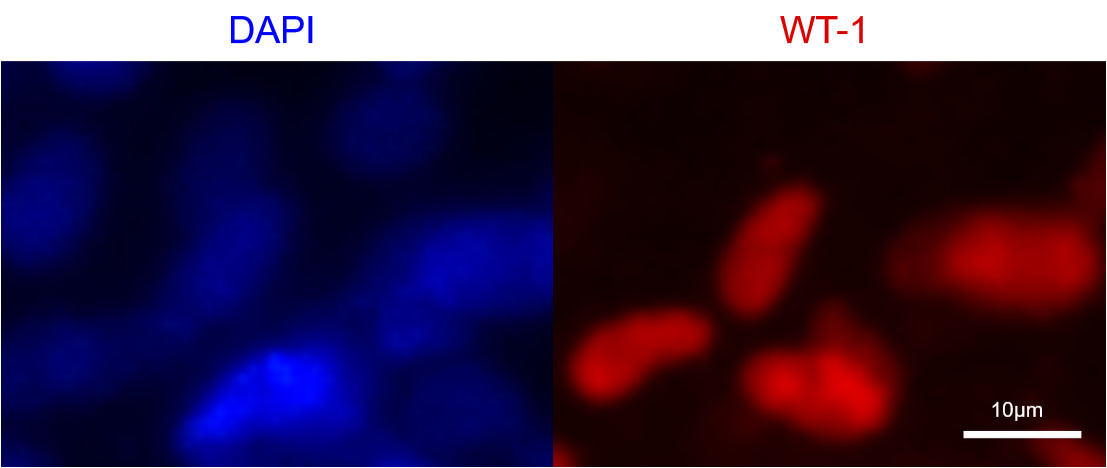
**Immunofluorescence staining of iPSC-derived cardiomyocytes**. Left: DAPI (blue)—nuclear staining; right: WT-1 (red)—cardiac-specific protein marker. DAPI, 4′,6-diamidino-2-phenylindole; WT-1, Wilms’ tumor Protein 1; iPSC, induced pluripotent stem cells. Scale bar: 10μm.

The efficiency of differentiating iPSCs into CMs has also improved from low to high; early attempts achieved minimal success rates of only 5–10%. However, by fine-tuning the regulation of pathways such as Wnt signaling and adopting a three-stage differentiation protocol, it is now possible to induce the differentiation of CMs with up to 95% purity using serum-free conditions and specific chemical factors alone [46, 47, 48]. Thus, this process leverages the staged activation and inhibition of Wnt signaling to precisely guide cells from mesodermal precursors to maturity, beating CMs and metabolic regulation to optimize the differentiation efficiency and cell purity further [49]. Fig. 3 shows CM differentiation following Wnt signaling pathway modulation and staining using an immunofluorescent antibody specific to the cardiac-specific marker Wilms’ tumor 1 antibody (WT-1) (Abcam, ab89901, Shanghai, China). Recent studies have shown that nanotextured platforms can further enhance the efficiency of iPSC differentiation into CMs [50]. Researchers have also analyzed the gene expression profiles of iPSCs cultured on different nano-topographies, thus enabling the identification of small-molecule drugs that can modulate differentiation efficiency.

In summary, current CTE has a robust foundation of cellular resources thanks to major advances in cell reprogramming and differentiation technologies, particularly when iPSCs are used.

### 3.2 Design and Preparation of Scaffolding Materials

Scaffolding materials play an important role in CTE. They serve as physical supports for cell attachment, proliferation, and differentiation, promoting tissue formation and functional recovery. With the rapid development of 3D bioprinting technology, the construction of personalized and precisely designed scaffolds has become feasible, enabling the creation of complex cardiac tissue structures [13, 51, 52].

Interactions between the scaffold and cells are pivotal during tissue regeneration. These interactions affect tissue maturation and the characteristic alignment of CMs along extracellular matrix (ECM) fibers. Unlike the unidirectional arrangement seen in skeletal muscle, the unique interlaced pattern of CMs forms a highly coordinated contraction network that ensures an effective cardiac pumping function. This special cell alignment endows cardiac muscle tissue with specific anisotropic mechanical behavior, thus highlighting the complex challenges in designing matching scaffold materials [53]. Therefore, CTE scaffolds need to meet a stringent set of criteria:

(1) Biocompatibility: ensures low immunogenicity post-implantation and reduces the risk of thrombosis.

(2) Degradability: capable of safe degradation via multiple bodily pathways, avoiding long-term residue.

(3) Mechanical strength: mimicking the elastic modulus of myocardial tissue (0.02–0.50 MPa) to maintain structural stability [54, 55, 56].

(4) Bioactivity: promoting cell adhesion, proliferation, and differentiation to accelerate tissue remodeling.

(5) Conductivity: supporting the synchronous contraction of CMs to maintain the electrophysiological characteristics necessary for cardiac pumping.

(6) Anisotropy: replicating the natural alignment of CMs to guide oriented cell growth and promote functional recovery [1].

These criteria are fundamental to ensuring that engineered scaffolds support and enhance the therapeutic potential of CTE.

#### 3.2.1 Natural Materials

Utilizing natural materials derived from the cardiac ECM is a highly rational strategy in CTE. These materials closely simulate the physiological conditions within the body and exhibit excellent biocompatibility and minimal immunogenicity, thereby providing an ideal microenvironment for cell growth. For the reasons given below, collagen and collagen-based hydrogels are the most widely utilized ECM components in CTE.

Approximately 75–90% of the cardiac ECM comprises collagen, which forms an ordered, anisotropic fibrous structure. This abundant natural resource makes extracting collagen on a large scale from various animal tissues feasible. Moreover, collagen and its scaffold materials possess outstanding biocompatibility and cross-linking properties, allowing them to provide an environment similar to the natural milieu of CMs and actively promote tissue reconstruction guided by cells [52].

However, collagen has limitations, including relatively low solubility at neutral pH and inadequate mechanical strength. To address these issues, collagen is often combined with other natural materials, including fibrin [57, 58], laminin [59], chitosan [60], and alginate [61], to enhance its performance. Given its ready acquisition and multi-functionality, collagen has become a preferred material for constructing cardiac tissues and developing cardiac patches [51].

This integrative approach ensures the engineered scaffolds not only support but also enhance the therapeutic potential of CTE, making collagen a cornerstone material in the field.

#### 3.2.2 Synthetic Polymer Materials

Synthetic polymer materials are favored for their non-toxicity, biodegradability, and high mechanical strength. Examples include polyethylene glycol (PEG) [62], polycaprolactone (PCL) [63, 64, 65], and polylactic acid (PLA) [66]. These polymer scaffold materials share the common attribute of being gradually remodeled and replaced by cells and the ECM over time [67]. Although artificial synthetic biomaterials may lag behind natural hydrogels in promoting cardiac tissue growth* in situ*, the biochemical properties, mechanical performance, and structural characteristics of these materials can be fine-tuned, thus providing new possibilities for precision medicine and tissue engineering [68].

Customizing these synthetic polymers offers significant advantages for tailoring materials to specific clinical needs, such as creating scaffolds with optimized porosity, degradation rates, and mechanical properties that closely mimic the native cardiac environment. This customization is crucial for advancing the field of CTE toward more effective therapeutic applications.

#### 3.2.3 Hybrid Materials

A focal point of CTE research has been integrating the advantages of natural materials with those of synthetic polymers, hybrid biomaterials, and advanced recombinant hydrogel systems. These materials combine the bioactivity of natural biomaterials with the structural flexibility of synthetic polymers, resulting in a unique application potential [69]. Subsequently, a recent study reported the successful development of a new type of biodegradable hybrid scaffold [70].

The scientific community is also actively exploring the application of recombinant hydrogels as platforms for CM cultures to emulate the natural alignment and complex structure of cardiac tissue [71]. Lee *et al*. [48] reported the design of conductive hydrogels that mimic the electrophysiological properties of cardiac tissue and promoted the regeneration process by providing the necessary mechanical and electrical stimuli, thereby guiding the repair of infarcted cardiac regions. Furthermore, the clinical potential of these conductive hydrogels extends beyond cardiac tissue to the regeneration process of other electrically active tissues, such as neural and muscular tissues.

Although recombinant hydrogels are promising for CTE, their clinical translation still faces several technical challenges. First, the long-term stability and biocompatibility of these materials must be optimized while ensuring they possess mechanical properties similar to those of natural cardiac tissue. Second, these materials must be effectively integrated with host cardiac tissue and internal vascularization must be achieved. Therefore, while recombinant hydrogels bring innovative solutions to CTE, further research and technological improvements are needed to overcome the remaining scientific and technical barriers before they can be clinically applied.

### 3.3 Tissue Engineering Construction Technologies

The core of CTE lies in integrating cells, biocompatible scaffold materials, and the necessary biological signals to create substitutes that mimic the structure and function of natural cardiac tissue [13]. Three-dimensional printing technology has emerged as a research hotspot due to its capabilities in macrostructural design and precise cell positioning. However, given the complexity of cardiac tissue structures, replicating the entire organ remains a significant challenge. Currently, 3D printing technologies and various biomaterial preparations are predominantly focused on producing acellular scaffolds or templates that can serve as foundational frameworks for cell growth, with less emphasis on direct cell printing [72].

Three-dimensional printing achieves the desired formation by adding layers of material, thus offering significantly enhanced manufacturing flexibility and personalization compared to traditional subtractive manufacturing techniques. Moreover, digital design and direct material conversion using this technology have created more options in the medical field [73]. This section will delve further into the specific applications and recent advances of 3D printing technology in CTE.

By leveraging 3D printing, researchers can tailor the architecture of scaffolds to match the intricate patterns of cardiac tissue, including the anisotropic arrangement of CMs and the vasculature network. This level of customization is critical for achieving proper cell alignment, tissue maturation, and integration of the engineered tissue with the host’s native tissue. While predominantly more challenging, direct cell printing allows the creation of living constructs with embedded cells, potentially leading to better integration and functionality following implantation. As this technology continues to evolve, further innovations can be expected in designing and fabricating cardiac tissues more closely resembling natural heart structures and functions.

#### 3.3.1 Inkjet and Extrusion Bioprinting

Inkjet bioprinting technology relies on diverse driving mechanisms, such as thermal energy, pressure, electric fields, or electromagnetic forces, to precisely deposit bio-ink droplets onto a plane. This innovative process replaces the “ink” in traditional printing with specialized “bio-inks” containing live cells, hydrogels, and other biologically active components, creating new fields in tissue engineering [74]. To ensure biocompatibility and functionality during the printing process, researchers have modified standard inkjet printing equipment to handle biomaterials, thus maintaining cellular viability and structural integrity [75].

Despite its cost-effectiveness and widespread use, thermal inkjet printing has several limitations, including imprecise control over droplet direction, frequent nozzle clogging, and potential cell damage due to high temperatures and strong mechanical forces. These factors threaten cell survival and limit the ability to build structures with a high cell density, which are essential for mimicking the complexity and functionality of cardiac tissue [76]. Hence, thermal inkjet printing is commonly used to produce acellular tissue engineering scaffolds. Because high cell viability and complex tissue structures are required in CTE, using gentler and more precise bioprinting strategies is particularly important.

Extrusion printing technology is one of the core methods used in tissue engineering since it enables the 3D assembly of cells and biomaterials through the precise application of mechanical force to push the bio-ink through a nozzle. This technique is particularly suited for constructing structurally complex tissue models with high cell density, such as those mimicking the properties of natural cardiac tissue in CTE. The absence of thermal processing reduces potential damage to cell viability, making this technology ideal for building large, complex biological structures such as those used in vascularized organ regeneration [13].

The introduction of multi-nozzle systems has further enhanced the capabilities of extrusion printing, increasing the speed of construction and the ability to integrate multiple cell types and biomaterials within a single structure, which is critical for replicating the heterogeneity found in natural tissues. Additionally, by fine-tuning parameters, such as the dispensing speed, pressure, and nozzle size, the precise deposition of cells and materials can be achieved while ensuring cell survival. This is crucial for maintaining cellular function and promoting tissue integration [77].

Despite the flexibility and cost-effectiveness of inkjet and extrusion printing in bioprinting, these methods share common challenges, particularly the risk of shear stress-induced damage to cells during the printing process. The high shear forces experienced when cells pass through narrow nozzles can lead to cellular damage or death, thereby impacting the functionality of the final printed structure [78]. Researchers have developed hydrogel formulations that respond dynamically to applied mechanical forces. These exhibit decreased viscosity with increased shear rate, effectively cushioning the shear stress experienced by cells and significantly improving cell survival rates and the overall quality of the printed structure [79].

Inkjet printing technology is cost-effective but requires more precise control of droplet direction, nozzle clogging, and limitations in constructing structures with a high cell density. Comparatively, extrusion printing technology is suitable for building complex structures, and non-thermal processing reduces cell damage. Multi-nozzle systems improve construction speed and diversity, although the high shear stress can damage cells. Through continuous technological innovation and advances in biomaterial science, both extrusion and inkjet printing technologies will become more efficient, biocompatible, and cell-friendly, thus creating increased possibilities in the field of tissue engineering [80].

#### 3.3.2 Light-Assisted Printing

Light-assisted bioprinting technology is another innovative branch of biomanufacturing that offers unique solutions for CTE. By precisely controlling light-induced polymerization reactions, this technique enables the three-dimensional spatial positioning of cells and biomaterials, thus presenting significant advantages for constructing complex biological structures [81].

Digital light processing (DLP) bioprinting utilizes digital projection technology to selectively cure liquid photopolymerizable bio-inks in the form of light spots, thereby creating structures with micron-level precision. This method has rapid prototyping capabilities and high resolution, typically ranging from 50 to 100 microns, making it suitable for rapidly manufacturing tissue models with intricate details. However, major challenges exist with DLP bioprinting, including its dependence on photopolymerizable materials and the potential adverse effects of ultraviolet (UV) light on cells. Subsequently, researchers are addressing these issues by adopting curing strategies that use visible light, thus helping to reduce cellular damage and maintain high cell viability [82, 83, 84].

Laser-assisted bioprinting (LAB) harnesses laser technology to achieve high precision and cell handling capabilities by precisely controlling the position of bio-ink droplets and the curing process [85]. LAB can handle individual cells with extremely high resolution, maintaining cell viability and sustaining high cell densities during construction. This is critical for applications such as CTE that require a high cell density. The speed and efficiency of LAB are also conducive to the mass production of complex biological structures, particularly in scenarios where precise cell alignment and restoration of tissue functionality are required [13].

Despite its outstanding resolution, biocompatibility, and efficiency, LAB nevertheless faces limitations related to material constraints and costs. Moreover, the reliance of LAB on photopolymerizable materials limits the range of available source materials. Although chemical modifications can expand this range, this adds to the complexity and expense of the process. Meanwhile, light-curing materials can circumvent common issues such as nozzle clogging and cell shear stress encountered in traditional extrusion printing [86].

In summary, light-assisted bioprinting technologies, including LAB, have opened new possibilities for CTE and other advanced biomanufacturing applications. Their high precision, efficiency, and cell compatibility make them powerful tools for constructing functional biological structures. Future research is likely to explore a broader range of biocompatible and photopolymerizable materials, optimize light processing to minimize cell damage, and strive to reduce costs to facilitate the clinical application of these technologies.

### 3.4 Biological Signaling and Regulation

Within the body, cardiac cells interact through a complex network of biological signals essential for maintaining the normal function of cardiac tissue. Simulating these biological signals in CTE is crucial for promoting tissue growth and functional recovery, thus representing a significant challenge and focal point. Signaling regulation can be achieved through controlled manipulation of the scaffold microenvironment, the addition of growth factors, and modification of the physical and chemical properties of the scaffold [13, 47].

#### 3.4.1 Controlling the Scaffold Microenvironment

One way to regulate biological signaling within a tissue-engineered construct is by carefully designing the scaffold’s microenvironment. This includes optimizing the scaffold’s porosity, pore size, and surface chemistry to promote cell attachment, proliferation, and differentiation. The scaffold should mimic the ECM to provide appropriate mechanical support and cues for cell behavior [77].

#### 3.4.2 Addition of Growth Factors

Growth factors play a critical role in regulating cell behavior and tissue development. By incorporating growth factors into the scaffold, it is possible to guide the formation of new tissue and to promote angiogenesis, which is essential for the vascularization of engineered tissue [13]. Examples of growth factors include vascular endothelial growth factor (VEGF) [87] for promoting blood vessel formation, fibroblast growth factor (FGF) for promoting cell proliferation and differentiation, and poly-3-hydroxyoctanoate (P[3HO]) for enhancing the mechanical properties of the patch [88, 89].

#### 3.4.3 Regulation of Physical and Chemical Properties

The physical and chemical properties of scaffolds, such as stiffness, elasticity, and surface characteristics, can affect cellular responses. Therefore, adjusting these properties helps to align cells correctly, which is particularly important for the anisotropic nature of cardiac tissue [1]. For example, Zhu *et al*. [90] used gold nanorods as rheological modifiers to adjust gelatin methacrylate (GelMA) bio-ink to mimic the morphological and mechanical features of natural tissue and induce the spread of cells. The adjusted gold nanorod–GelMA hydrogel had Young’s modulus of 4.2 ± 0.3 kPa, which was higher than the original GelMA hydrogel (3.75 ± 0.15 kPa), thus making it more suitable for cardiac tissue implantation. Moreover, CMs on the gold nanorod-integrated GelMA scaffold began synchronous rhythmic contractions on day 2—much earlier than the CMs cultured on the original GelMA hydrogel (day 5). Lei *et al*. [14] produced microscale PCL fibers with an average size of 9.5 µm using melt-based electrohydrodynamic (EHD) printing. These were used to mimic cardiac collagenous fibers, which guided layer-specific cell orientations.

**Fig. 4.**
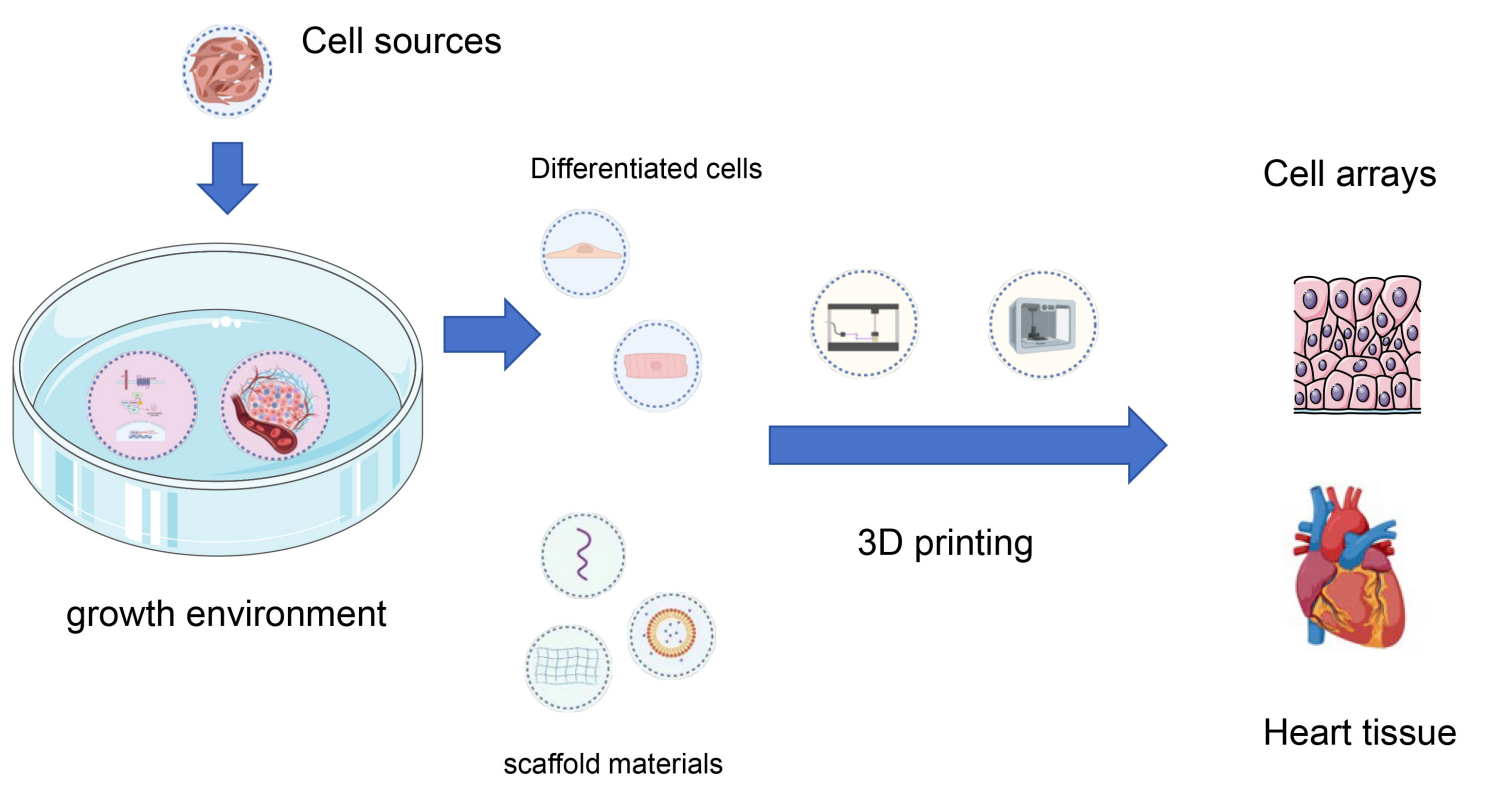
**The roles of the four components in cardiac tissue engineering**. 3D, three-dimensional.

**Fig. 5.**
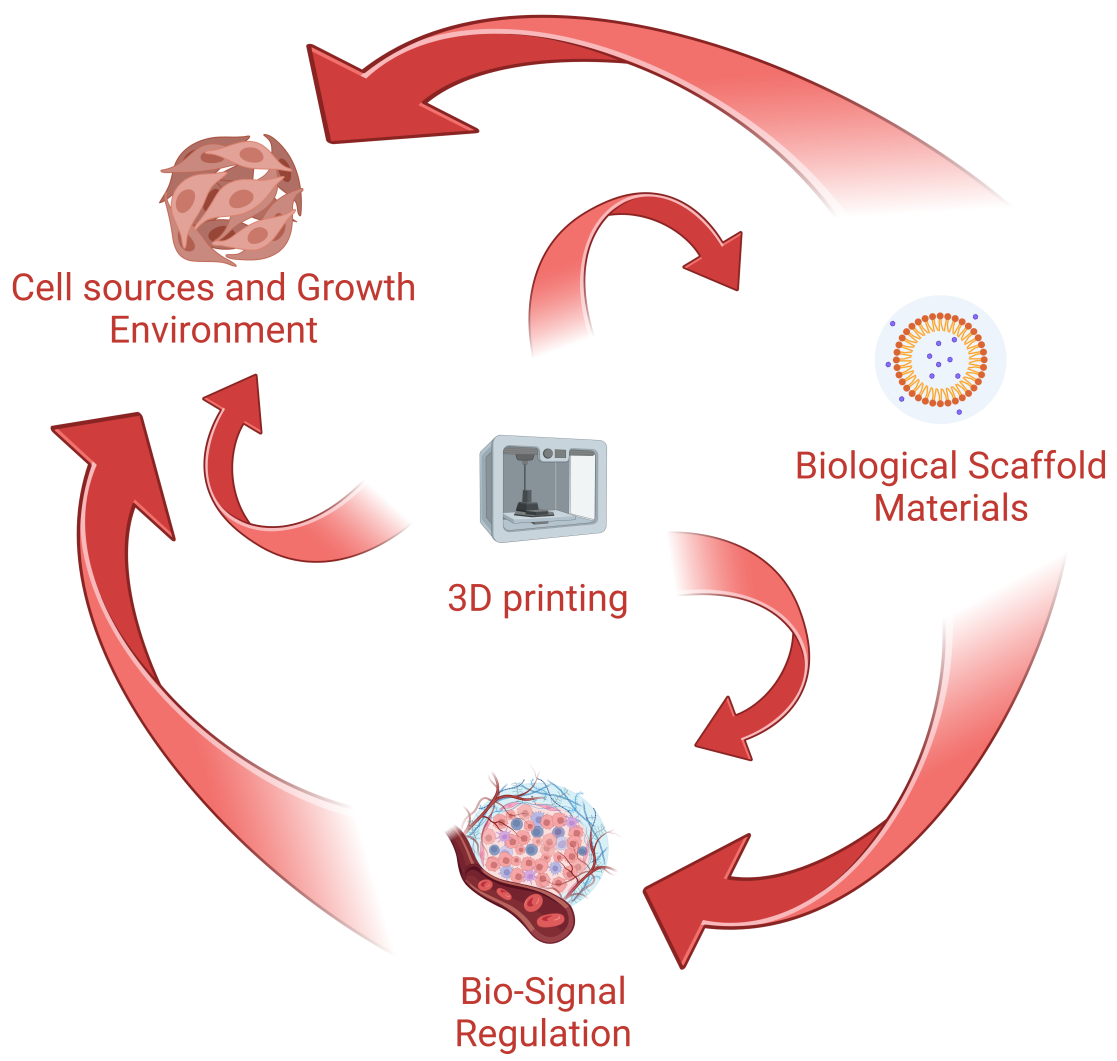
**Signal pathways are crucial in cell growth and differentiation**. By activating specific signal pathways, it is possible to guide stem cells to differentiate into particular cell types, such as CMs [92]. The physical and chemical properties of scaffold materials, such as porosity, biocompatibility, and mechanical strength, significantly influence cell adhesion, proliferation, and differentiation. For example, porous scaffold materials can promote cell infiltration and distribution [93]. Three-dimensional printing technology enables the design and fabrication of scaffold materials with specific chemical and physical characteristics. These properties can activate or inhibit particular signal pathways, thereby modulating cell behavior. For instance, scaffold materials containing specific growth factors can enhance cell proliferation and differentiation [94]. 3D, three-dimensional; CMs, cardiomyocytes. The Figure is created by BioRender.

Additionally, Feng *et al*. [91] utilized shape memory polymers (SMPs) to create self-adhesive, conductive cardiac patches that promoted the conduction of cardiac electrical signals and improved the function of myocardial regions. The above studies demonstrate the feasibility of improving the alignment and function of cells by adjusting the physical and chemical properties of scaffolds. The efficacy of engineered tissues can also be enhanced by introducing biologically active molecules that interact with cells and regulate their behavior. By integrating these approaches and materials, CTE can achieve cardiac repair and replacement based on biomaterials, thus offering novel strategies for treating cardiovascular diseases. Creating functional cardiac tissue that integrates well with the host environment and promotes healing could revolutionize current treatment paradigms for heart-related conditions. Fig. 4 illustrates the basic process of CTE; meanwhile, Fig. 5 (Ref. [92, 93, 94]) illustrates the interactions among four key areas in cardiac tissue engineering.

## 4. Discussion

CTE is a pioneering endeavor within biomedical engineering that aims to address the growing disparity between scarce organ transplant resources and increasing demand; this field focuses on two main technological paradigms: scaffold-dependent and scaffold-free bioprinting. These paradigms provide a scientific framework and practical pathways for tissue regeneration and functional restoration.

### 4.1 Scaffold-Free Bioprinting

Scaffold-free bioprinting technology dispenses with traditional scaffold structures and instead utilizes bioprinting techniques to arrange living cells precisely within an *in vitro* environment. This promotes the self-assembly of cells into structures such as cardiac patches, spheroids, and even organ-like prototypes. Moreover, scaffold-free bioprinting technology significantly expands the boundaries of tissue engineering by creating conditions for constructing more natural tissue morphologies and functionalities. Table 3 (Ref. [17, 18, 19, 20, 21, 22, 23, 24]) presents some representative outcomes of scaffold-free printing techniques.

**Table 3.** **Summary of scaffold-free bioprinting approaches for cardiac tissue engineering**.

Researchers	Publication year	Method	Bio-ink	Shape (dimensions)	Cell survival rate	Animal model	Significance
Arai *et al*. [17]	2018	Extrusion printing	hiPSC–CMs, HUVECs, HDFs	Tubular (N/A)	Apoptotic cells were present in the central region	N/A	Produced an *in vitro* model closer to physiological conditions for heart disease treatment and drug screening
Maiullari *et al*. [18]	2018	Extrusion printing	HUVECs, hiPSC–CMs	Cardiac-like structure (8 mm × 8 mm × 1 mm)	N/A	N/A	Manufactured vasculature-rich cardiac tissue to promote cardiac tissue regeneration and established a research model for cardiovascular diseases
Anil Kumar *et al*. [19]	2019	Extrusion printing	hiPSC–CMs, CFs	Cardiac-like structure (1 cm × 1 cm × 500 µm)	92 ± 3%	N/A	Photocuring printing helps to develop tissue-engineered cardiac patches, especially in constructing biological activity and good cell integration models
Yu *et al*. [20]	2019	DLP	dECM, hiPSC–CMs	Patch with a striped pattern (3 mm × 3 mm × 250 µm)	High cell viability	N/A	Presented a novel approach for the rapid construction of biomimetic human tissues possessing tissue-specific biochemical constituents, microscale microarchitecture, and tailorable modulus
Yeung *et al*. [21]	2019	Extrusion printing	hiPSC–CMs, HDFs, HUVECs	Patch (3.6 mm × 3.6 mm × 350–400 µm)	94.7 ± 2.78%	Rat	Promoting cardiac tissue regeneration and angiogenesis, reducing fibrosis formation, promising for heart failure treatment
Lou *et al*. [22]	2023	Extrusion printing	hiPSC-derived CMs, smooth muscle cells, endothelial cells, fibroblasts	Patch (1 cm × 1 cm × 2 mm)	>95%	Mouse	Exploring multi-cell strategies for cardiac repair potential, promoting cardiac function recovery
Noor *et al*. [23]	2019	Extrusion printing	hiPSC–CMs, hiPSC–ECs	Patch (3.7 cm × 2.8 cm × 2 mm), cardiac-like structure (height: 20 mm; diameter: 14 mm)	100%	N/A	Innovative use of autologous fat-derived ipsc and ECM to construct an allograft-free cardiac patch
Pretorius *et al*. [24]	2021	Layer-by-layer assembly	hiPsC–CMs, CFs, hiPSC–ECs	Patch (thickness >2 mm)	>94%	N/A	Producing clinically relevant size and thickness of engineered cardiac tissue, maintaining high cell viability and electrophysiological properties

hiPSC–CMs, human induced pluripotent stem cell-derived cardiomyocytes; HUVECs, human umbilical vein endothelial cells; HDFs, human dermal fibroblasts; CFs, cardiac fibroblasts; ECs, endothelial cells; dECM, decellularized extracellular matrix; N/A, not applicable; ECM, extracellular matrix; DLP, digital light processing.

#### 4.1.1 *In Vitro* Research Models

Maiullari *et al*. [18] utilized the precise control offered by microfluidic printing technology to arrange human induced pluripotent stem cells (hiPSC)–CMs and human umbilical vein endothelial cells (HUVECs) as bio-inks within a printed structure. This approach allowed simulation of the complex cellular composition and alignment found in cardiac tissue, resulting in the directed orientation of CMs along the printed fibers. This promoted the ordered structure of tissues and enhanced their function. Overall, the model applied by Maiullari *et al*. [18] demonstrates the feasibility of constructing vascularized cardiac tissues, contributes to advancing cardiac regenerative medicine, and is a valuable tool for cardiovascular disease research. Anil Kumar *et al*. [19] developed an innovative bio-ink composed of fibrin–gelatin composite cross-linked with visible light and combined this with hiPSC–CMs and primary human cardiac fibroblasts (CFs). The unique aspect of this bio-ink is the combination of fibrin’s excellent biocompatibility with gelatin’s photocurable properties to provide a stable and cell-friendly environment for the construction of 3D cardiac tissue models. This advance facilitates the creation of tissue constructs with cell specificity and complex organizational structures, thereby enhancing the simulation capabilities in cardiac tissue research. Yu *et al*. [20] utilized decellularized extracellular matrix (dECM) and hiPSC–CMs as bio-inks to fabricate cellular patches with a 60-micron-wide, 60-micron-spaced striped pattern measuring 3 mm × 3 mm × 250 µm, using a DLP-based 3D bioprinting platform. Compared to extrusion printing, light-assisted printing technology exhibits significantly higher resolution; moreover, while the resolution of dECM bio-inks in extrusion printing is typically no less than 100 µm, light-assisted printing can achieve lines as fine as 30 µm. Furthermore, light-assisted printing technology enables the facile adjustment of the mechanical properties of the final printed product by simply modifying the exposure time without altering the bio-ink formulation.

#### 4.1.2 *In Vivo* Animal Studies

Yeung *et al*. [21] conducted an *in vivo* study using a cardiac patch comprising 70% hiPSC–CMs, 15% human dermal fibroblasts (HDFs), and 15% HUVECs. These cardiac spheres were 3D-printed into a patch and then implanted into a rat model of myocardial infarction to assess their regenerative potential in a living organism. Rats that received the 3D-printed cardiac patch showed increased angiogenesis and a reduced scar area in the damaged heart region compared to the control group that did not receive the patch. At 4 weeks post-surgery, the average percentage of scar area in the patch group (10.6% ± 5.1%) was significantly lower than in the control group (19.39% ± 8.1%). Meanwhile, although there was also a trend toward improved cardiac function, this was not statistically significant in the short term. Nonetheless, this scaffold-free 3D-printed cardiac patch showed that it could promote the regeneration and vascularization of cardiac tissue and reduce scar tissue formation, thus offering promise as a novel approach for treating heart failure. Lou *et al*. [22] constructed a cardiac patch by combining four types of cardiac cells derived from human pluripotent stem cells: CMs, smooth muscle cells, endothelial cells (ECs), and fibroblasts. The patch by Lou *et al*. [22] was designed to promote the recovery of cardiac function in a mouse model of cardiac injury. Further building on the work of Yeung *et al*. [21], which showed that a 3-cell-type patch improved cardiac function in a rat model, the study by Lou *et al*. [22] investigated the potential of a multi-cell strategy for cardiac repair by incorporating smooth muscle cells. The average percentage of scar area in their 4-cell patch group (22.72% ± 0.98) at 4 weeks post-surgery was significantly lower than in the 3-cell patch group (39.23% ± 4.28). Hence, the inclusion of additional cell types may enhance the repair capacity of the patch, potentially leading to better recovery of cardiac function.

#### 4.1.3 Innovative Strategies

Noor *et al*. [23] introduced an innovative strategy that uses adipose tissue as a core material. A small sample of adipose tissue was harvested, and the cells were reprogrammed into pluripotent stem cells and then differentiated into CMs and ECs. The ECM derived from the adipose tissue was processed into a hydrogel. The cells were subsequently mixed with the hydrogel to form two distinct bio-inks: one for printing myocardial tissue and another for embedding the vasculature. Using mathematical modeling to optimize the vascular structure of the patch for improved oxygen transport efficiency, the researchers then employed 3D printing technology to construct a patch containing myocardial tissue and embedded vasculature. This approach leverages the unique properties of adipose-derived materials to create a more biocompatible and functional cardiac patch. Pretorius *et al*. [24] proposed an innovative CTE strategy that uses a layer-by-layer (LbL) assembly method to produce large, thick human myocardial patches. These authors differentiated iPSCs into CMs, ECs, and CFs. Moreover, they mixed the CMs with a fibrin-based matrix and placed this mixture into a polycarbonate mold to form the first layer. On top of the first layer, they deposited a second layer of ECs, followed by a third layer of CFs. The LbL method was used to construct engineered cardiac tissue with a thickness of up to 2.12 mm. These tissues exhibited a high cell survival rate (<6% cell necrosis) over four weeks of *in vitro* culturing while maintaining good electrophysiological characteristics and tissue stability. The study successfully produced engineered cardiac tissue with clinically relevant dimensions and thickness. This also demonstrated superior electrophysiological performance and structural integrity, providing significant insights for cardiac regenerative medicine.

### 4.2 Scaffold-Dependent Bioprinting

Scaffold-dependent bioprinting primarily involves attaching stem cells to carefully engineered scaffolds that can be natural or synthetic. Within a precisely controlled microenvironment, the cells undergo ordered proliferation and differentiation to gradually form functional cardiac tissues, complex vascular networks, and valve structures. Over the past few decades, this technology has shown significant success in experimental settings and early clinical applications, thus advancing CTE. Table 4 (Ref. [25, 26, 27]) lists the representative outcomes of scaffold-dependent printing techniques.

**Table 4.** **Summary of scaffold-based bioprinting approaches for cardiac tissue engineering**.

Researchers	Year	Method	Bio-ink	Shape (dimensions)	Significance
Tsukamoto *et al*. [25]	2020	HBC–gelatin framework-controlled 3D printing	HBC, hiPSC-CMs, CFs	Oriented vascular networks (N/A)	Provides a model closer to natural cardiac tissue that is useful for drug screening and heart disease treatment research
Miller *et al*. [26]	2021	Micro-stereolithography printing	hiPSC–CMs, CFs with methacrylate gelatin	Microtissues with fine wire-like scaffold structures (N/A)	Demonstrates the potential of 3D printing technology in generating complex cardiac tissue structures, valuable for cardiovascular disease research and drug screening
Sridharan *et al*. [27]	2021	Electrospinning	Polyethylene glycol, gelatin, hiPSC–CMs	Composite material scaffolds (N/A)	Simulating the microenvironment of heart tissue, promoting cell survival, proliferation, and guiding cells to differentiate into functional tissues, used for heart tissue regeneration and repair

HBC, hydroxybutyl chitosan; hiPSC–CMs, human induced pluripotent stem cell-derived cardiomyocytes; CFs, cardiac fibroblasts; N/A, not available; 3D, three-dimensional.

Tsukamoto *et al*. [25] used hydroxybutyl chitosan (HBC) as a material to create 3D-oriented cardiac tissue via 3D printing technology. Initially, they constructed a gel framework to guide the deposition of cells. Subsequently, they employed the LbL technique to encapsulate hiPSC–CMs and CFs within an outer film placed inside the printed HBC gel. The results showed that cells were aligned within the 3D structure and exhibited superior tissue contraction performance compared to non-aligned tissues. The authors established a vascular network by co-culturing these structures with human microvascular ECs, which is crucial for maintaining long-term tissue viability. The printed cardiac tissue featured aligned CMs and CFs and an integrated vascular network, thereby mimicking the cardiac microenvironment. This highly biomimetic model offers a platform for cardiac research and therapy, advancing our ability to create realistic models for studying and treating heart conditions.

Miller *et al*. [26] applied innovative bioprinting technology using hiPSC–CMs, CFs, and GelMA as the bio-ink. They used a micro-continuous optical printing system to control the UV curing process more precisely, constructing detailed myocardial tissue models. These printed micro-tissues rapidly exhibited the characteristic cardiac contractions and contained tightly packed cells with good viability. Subsequent gene analysis also confirmed cell maturity and enhanced expression of marker genes. Thus, this method demonstrates the ability of 3D printing to construct complex tissues while also providing an advanced model system for cardiovascular disease research, drug screening, and studies of cardiac tissue repair.

Sridharan *et al*. [27] described the fabrication of an aligned, coaxial nanofiber scaffold composed of a PCL core and a gelatin shell, along with an effective method for seeding and orienting hiPSC–CMs on these scaffolds. This approach aimed to provide a platform for creating functional “cardiac patches” that can be used for cardiac repair and *in vitro* 3D cardiac tissue models to evaluate the efficacy and cardiotoxicity of cardiovascular drugs.

### 4.3 Perspectives and Challenges

The rapid advances in CTE, particularly the breakthroughs in scaffold-free and scaffold-dependent bioprinting technologies, highlight the significant progress in addressing the challenges of treating heart disease. Beauchamp *et al*. [95] demonstrated the importance of physiological relevance using 3D co-culture cardiac spheroid models that provide a more accurate platform for drug screening and personalized medical interventions. Meanwhile, Arai *et al*. [17] refined the *in vitro* replication of complex cardiac structures by printing oriented cardiac tubular structures. This offers new perspectives for understanding heart development and disease mechanisms while suggesting potentially novel pathways for tissue repair. Despite these notable achievements, the field of CTE still faces several challenges, such as those outlined below.

### 4.4 Tissue Vascularization

Effective vascularization is critical for the long-term survival of transplanted tissues, as it ensures the delivery of oxygen and nutrients and the removal of metabolic waste. The formation of networks within printed structures that mimic the natural vasculature and promote the effective connection of new vessels to the host’s vascular system currently presents a major challenge. To ensure cardiac tissues survive and function *in vivo*, they must possess a good vascular network to supply oxygen and nutrients; however, the maximum nutrient/oxygen diffusion distance for cells without vascularization is approximately 100–200 µm [9]. Thus, several research strategies are attempting to increase the vasculature, including through the use of 3D microchannel “AngioChip” scaffolds to support the assembly of mm-thick vascularized cardiac tissues [96], the development of multi-component hydrogel bio-inks [97], and the direct printing of vascular systems [98]. However, these methods still require further refinement to meet clinical application standards.

### 4.5 Material and Technology Optimization

Light-assisted printing technologies such as DLP and LAB have enabled higher resolution and better preservation of cell viability. However, the limited range of available photosensitive materials and their potential cytotoxicity currently impede the more widespread application of this technology. Therefore, key directions for future research include developing more photosensitive materials that are harmless to cells and optimizing the printing process to minimize cell damage [82].

Another important research direction is the development of bio-inks. Indeed, the ideal bio-ink should be printable, biologically active, biodegradable, stable, affordable, suitable for commercialization, and have appropriate regulatory guidelines for clinical use [96]. However, existing materials require improvement in one or more areas. Hydrogels are a commonly used material for bioprinting in cardiovascular applications because they provide good support for cells. However, single-component hydrogels do not fully replicate the natural environment of cardiac cells. Therefore, the development of composite bio-inks remains a priority for future research.

### 4.6 Long-Term Functionality and Animal Experiments

The key factors in building viable cardiac tissues after printing are ensuring a high cell survival rate and maintaining long-term functionality. Meanwhile, optimizing the survival environment of cells during and after printing requires long-term studies to evaluate cell vitality, phenotypic changes, and biological functions after printing. Most studies have been short-term experiments that do not include animal models. Hence, incorporating *in vivo* experiments into future research could better assess the biological safety and fidelity of printed models, thus providing solid evidence for future clinical trials.

### 4.7 Four-Dimensional Printing

Four-dimensional printing has attracted significant attention from the research community. Four-dimensional printing combines 3D printing technology with smart materials that respond to external stimuli (e.g., heat, humidity, light, pH), causing the shape, properties, or function to evolve, such as self-folding, drug release, or monitoring [99, 100]. Although the four-dimensional (4D) printing of cardiac tissue is still in the early stages, we speculate its development will be positive for CTE.

## 5. Conclusions

To achieve further advances in CTE, 3D printing technologies must continue to be optimized. In particular, more biocompatible and photocurable materials should be sought, while more precise printing devices should also be developed to improve cell survival and the complexity of tissue constructs. Additionally, there should be a strong emphasis on simulating the cardiac microenvironment, including more precise cell interaction and vascularization strategies and further research into personalized treatments using autologous cells. Finally, additional long-term animal and preclinical studies will ensure the biosafety and functionality of engineered cardiac tissues, which should facilitate their early adoption in clinical settings and bring new hope for treating patients with heart diseases.

## Availability of Data and Materials

All data and materials were from published researches.

## Supplementary Material

Supplementary material associated with this article can be found, in the online version, at https://doi.org/10.31083/RCM26697.
